# Supplementary material for: Are carbon emissions trading and green financial instruments synergistic? -Comprehensive quantitative research based on content analysis
Source: PLoS One. 2024 Mar 7;19(3):e0298601. doi: 10.1371/journal.pone.0298601 (PMC10919601; doi:10.1371/journal.pone.0298601)
Supplement: S1 Data — (ZIP) [file pone.0298601.s001.zip › Data/地级市Z_F工作报告.zip!/║┌┴·╜¡/╝╤─╛╦╣╩╨/└┤╫╘╣1⁄2╓┌║┼í╛┬φ┐╦╩2╛▌═°í┐_2014.docx]

各位代表：

现在，我代表市人民政府向大会作工作报告，请予审议，并请各位政协委员和列席会议的同志提出意见。

一、2013年工作回顾

2013年，在省委、省政府和市委的正确领导下，我们团结带领全市人民，在宏观经济增速趋缓的不利条件下，充分发挥四大优势，坚定不移地推进九大战略任务，较好地完成了市十五届人大二次会议确定的各项目标。预计全市地区生产总值完成735亿元，同比增长10%；公共财政预算收入完成41.8亿元，同比增长17.6%，市本级公共财政预算收入完成28.7亿元，同比增长16.7%。固定资产投资实现479.7亿元，同比增长30%。

（一）产业项目攻坚成效显著。全年开复工投资千万元以上产业项目660个，同比增加151个。其中投资亿元以上项目125个，同比增加28个；投资10亿元以上项目10个，同比增加2个。预计完成投资340亿元，同比增长54%。农副产品加工、装备制造、现代服务业和对俄贸易及加工四大类项目占全市项目总数的69.2%，占产业项目投资总额的82.9%，项目结构更加优化，主导产业优势更加突出。哈佳快速铁路、同江中俄跨江铁路大桥等重大基础设施项目推进效果明显。全年国内招商引资到位资金360亿元，同比增长30%，其中利用省外资金266.5亿元，同比增长46%；实际利用外资2.1亿美元，同比增长15%。重点建设的10个产业园区共获批土地64.7平方公里，基础设施建设累计投入61亿元，建设投资千万元以上产业项目271个，完成投资263亿元，7个园区列入省级产业园区。

（二）工业经济运行状况良好。继续坚持抓大不放小，新增规模以上企业107户、私营企业1474户，全市规模以上工业增加值实现114亿元，同比增长16.5%。企业自主创新步伐加快。新建企业技术研发机构5个，新认定国家高新技术企业5户、科技型中小企业20户、省级高新技术产品25个。企业应用类科技成果转化率超过75%，全年高新技术产业产值实现240亿元，同比增长20%。

（三）现代农业发展势头强劲。水稻、玉米两大高效作物占总播种面积的85.7%，同比提高7个百分点。蔬菜种植和林下经济规模持续扩大，水稻育秧大棚二次利用效果明显。新建大型农机合作社11个，农机总动力超过360万千瓦，综合农业机械化程度达到 91.7%。创新农业经营主体，新增各类合作经济组织761个、家庭农场336个，土地流转面积373.1万亩，同比增长22.6%。新开工建设投资千万元以上的农副产品加工项目117个，规模以上龙头企业达到163户，引领15万农户参与产业化经营。桦川大米、富锦大豆、桦南白瓜成为国家注册地理标志产品。农业基础设施建设完成投资7.16亿元，新增灌溉能力30万亩。粮食总产实现“十连增”。

（四）开放合作优势持续放大。深入开展与俄、韩、德、意等地方政府和国内友好城市间高层互访，对外合作拓展深化。预计外贸进出口总额实现22.5亿美元，其中对俄进出口总额实现11.7亿美元。中俄（佳木斯）农机展洽会升级为国家级展会，全国唯一的农作物收割机械设备质量监督检验中心通过国家验收，正式启动运营。佳木斯机场客流增量列全省支线机场首位。全市社会消费品零售总额同比增长14.5%，旅游总收入同比增长18.6%。交通银行、哈尔滨银行在我市相继建立分行，小额贷款公司实现县域覆盖。截至2013年11月末，各类金融机构存款余额分别同比增加107.7亿元和81.9亿元，存贷比提高1.06个百分点。

（五）城乡人居环境明显改善。城市控详规划实现主城区全覆盖。长安路、胜利路西延和长安东路拓宽改造等重点工程全部完成，解放路友谊立交桥工程实现通车，打通了重要交通节点，城市路网进一步优化。城区拆除违建34万平方米，整治裸地86万平方米，主要街路机械化清扫率超过80%，清雪机械化程度在全省率先达到80%，被评为全省清雪A类城市。全市新建改造热源新增供热能力687万平方米，完成既有建筑节能改造330.9万平方米，新增天然气用户2.9万户。全市7个整乡（镇）推进城镇化工作试点，完成投资8.9亿元，建成新型农村社区70个，65个村进行了整村改造。完成松花江水污染防治和污染减排项目48个，佳木斯出境断面水质达到国家考核标准。化学需氧量、二氧化硫排放量提前完成“十二五”减排任务。全市森林覆盖率、市区绿化覆盖率分别达到15.5%和41.1%，正式被命名为国家园林城市。

（六）惠民利民措施有效落实。全市民生投入110亿元，同比增长15.8%，确定的30件民生实事全部完成年度任务。全市实现新就业8.5万人，创业带动就业1.9万人，城镇登记失业率控制在4.3%以内。转移农村富余劳动力36.4万人次，实现劳务收入38.6亿元，超额完成年度任务。预计城镇居民人均可支配收入实现17590元，同比增长13%；农民人均纯收入实现11100元，同比增长13%。社会保险扩面4.68万人，实现医疗保险市级统筹。离退休职工人均增加基本养老金239元。城市低保标准由月人均310元提高到376元，农村低保标准由年人均2000元提高到2181元，均高于全省规定标准。新农合参合率99.79%，人均补助标准由240元提高到280元。住房公积金归集额10亿元，发放个人住房贷款6亿元。市本级投入1.2亿元维修改造资金，中小学校园环境、供热条件明显改善。市直三家医院实行先看病、后结算，六家医院实行低保人群一站式服务。全市996名村医工资纳入财政预算。快乐舞步健身操引领全国群体健身活动时尚。全市改造棚户区63.9万平方米，建设廉租房、公租房1500套，改造农村泥草房1.7万户，农村砖瓦化率达到77%。完成灾后维修、重建房屋总量68%的任务。清真寺建成交付使用。全面开展食品药品安全专项整治，取缔居民物业小区自备井，调整优化公交线路。完善社会矛盾调处机制，年度梳理的36件重点积案全部化解，本级信访立案办结率100%。“打防控”一体化建设扎实推进，“两抢一盗”发案率稳步下降。全年无重特大安全生产事故。统计、审计、气象、边防、人口计生等工作得到加强，妇女儿童、红十字、残联等工作扎实推进。

一年来，我们以建设为民务实清廉政府为目标，自觉接受人大法律监督、政协民主监督、社会舆论监督，主动听取民主党派、工商联和社会各界意见、建议，年度人大代表建议和政协提案办结率实现100%。深入开展转作风提效能活动，行政监察、政务公开、电子政务、政府诚信工作取得新进展。向城区下放行政权力565项，各类审批事项限时办结率达到100%。认真抓好重点领域、重要环节的监管和审计，严格控制公务消费，全面启动市直机关公务用车改革，“四项经费”支出实现零增长。

我们坚持以人为本，科学应对黑龙江、松花江特大洪水灾害。在党中央、国务院亲切关怀下，在省委、省政府和市委坚强领导下，在驻军和武警部队大力支持下，组织全市参战军民同心协力、顽强拼搏，赢得了抗洪抢险工作的全面胜利，确保了无人员因灾伤亡。

各位代表，过去的一年，面对宏观形势的严峻挑战，面对转型发展的迫切需要，全市上下发挥聪明才智，奋力开拓进取，取得了来之不易的发展成果。在此，我代表市人民政府，向全市人民，向各民主党派、工商联、人民团体，向驻佳部队和武警官兵，向中省直驻佳单位及所有关心、支持佳木斯发展的各界人士以及海内外佳木斯人表示崇高的敬意和衷心的感谢！

在总结成绩的同时，我们也清醒地看到发展中还存在一些问题和矛盾，主要表现在：经济总量不大、质量不优、主导产业不强；产业项目进展不平衡，个别县区、市直部门推进力度不大；受宏观经济形势影响，地区生产总值、公共财政预算收入等指标未能完成计划任务；百姓反映强烈的供热、供水等问题还没有彻底解决，居民回迁和房屋产权证照办理工作进展不大，农村环境综合整治成效不明显；行政权力清理、下放和承接不到位，个别部门抓工作落实力度不够，等等。对这些问题，我们将高度重视、认真解决。

二、2014年工作安排

2014年，是我市全面完成“十二五”规划至关重要的一年，我们要准确把握发展的阶段特征，有效应对经济增长的下行压力、瓶颈制约加剧带来的攻坚压力，坚持稳中求进，进中求好，改革创新，以职能的转变适应形势的新变化，以更高的追求引领经济社会的新发展。

今年政府工作指导思想是：以党的十八大和十八届三中全会精神为指导，贯彻落实省委、省政府突出“五大规划”发展战略的安排部署，按照市委十二届四次全会确定的目标任务，围绕科学发展主题，以改革创新为动力，继续突出四大优势，扎实推进九大战略任务，积极深化重点领域改革，切实提高经济发展速度和质量，着力改善民生，完善社会治理体系，提高社会治理水平，促进经济健康快速发展、社会和谐稳定。

今年全市经济社会发展主要目标是：地区生产总值增长10%以上，全社会固定资产投资增长30%以上，公共财政预算收入增长10%以上，城镇居民人均可支配收入和农村居民人均纯收入均增长11%以上，城乡建设、民生保障、生态保护等工作进一步加强，社会公共服务和社会管理水平明显提升。

（一）全力以赴，推进产业项目建设。按照省委、省政府十大重点产业项目建设的部署，聚焦政策、聚集资源、聚合力量，充分发挥四大优势，构建主导产业体系，启动实施新一轮产业项目三年攻坚。（1）发挥现代农业优势，围绕延伸粮食、畜禽、水产、山野土特产品等农副产品精深加工产业链条，在推动农业生产投入品、食品加工业扩大规模、提高档次、打造品牌方面上项目。（2）发挥工业基础优势，围绕装备制造业配套化、集群化发展及现有企业技术改造，在做大做强农机、电机、煤机产业，促进建材、造纸、医药、化工、纺织等传统优势产业提档升级，推动新兴产业扩张发展方面上项目。（3）发挥沿边开放优势，围绕黑龙江和内蒙古东北部地区沿边开发开放规划的战略布局，在以跨境物流带动对俄经贸合作、基地建设和加工业发展方面上项目。（4）发挥中心城市优势，围绕完善服务功能，提升服务业态，在做优现代服务业，提高城市综合承载力、竞争力和吸引力方面上项目。突出项目建设质量，全年完成产业项目投资同比增长35%以上，新谋划储备产业项目300项，开复工600项，其中新开工300项，竣工投产200项。力争哈佳快速铁路、同江中俄跨江铁路大桥尽快开工。改进招商方式，充分对接重点区域名企、商会、社团和域外佳木斯籍知名人士，加大引资、引技、引业态力度。全市国内招商引资到位资金同比增长20%以上，其中利用省外资金同比增长30%以上；实际利用外资同比增长15%以上。加强佳木斯高新技术产业开发区建设，扩大园区面积，完善管理服务，吸引规模大、成长性强的企业入区发展，打造集聚效应，积极申报和创建国家级开发区。

（二）多措并举，提升工业经济质量。落实省工业经济稳定增长新“17条”措施，支持工业经济加快发展。全市工业实现销售收入同比增长12%以上；实现增加值同比增长11%以上。充分发挥中小企业服务中心作用，重点加大对初创期、成长期中小微企业扶持力度，促其上规模、上层次。全市新增规模以上工业企业60户。加快实施创新驱动，以佳木斯大学等高校院所为依托，搭建产学研合作服务平台，为企业创新、成果转化提供科技支撑，高新技术产业产值同比增长20%以上。依托地方优势企业，建立产业技术创新战略联盟，推动机械制造、农副产品和绿色食品加工等特色产业集群化发展。

（三）深化改革，加快现代农业发展。以确保国家粮食安全、持续增加农民收入为目标，抓住“两大平原”现代农业综合配套改革试验的重大机遇，大胆试、大胆闯，以务实的试点成效，争取政策支持，释放改革红利。构建新型农业经营体系。把创新农业生产经营主体作为现代农业综合配套改革的突破口，发展家庭农场100个、专业大户200户，土地流转规模410万亩以上。加大农机具扶持力度，鼓励农民创办专业合作社，提高农业生产集约化、专业化、组织化、社会化程度和市场竞争力。先行先试，推进农民承包经营权流转，争取建立农村产权流转交易市场。加快农产品市场体系建设。提高我市农产品在全国实体市场及网络电商领域的知名度、认可度，着力打造地方优质水稻、山特产品及畜产品国内国际中高端市场消费品牌。调整农业产业结构。新增水稻、玉米种植面积100万亩，推进畜牧、水产、林下经济、绿色特色农业规模化、产业化、标准化、生态化发展。强化基础设施建设。加快水毁工程修复进度和灌排水利工程建设，在提高抗风险能力同时，提高农业水质，确保农作物增产优质。

（四）内引外联，彰显区域中心活力。推进万达广场、港龙东方城等城市综合体建设，同步发展电子商务、网络购物、服务外包等新型业态，加快形成与城市定位相适应的商业服务体系，引领周边消费市场，社会消费品零售总额同比增长13.5%以上。加大宣传力度，完善旅游基础设施，积极发展特色旅游，旅游收入同比增长18%以上。积极发展信息服务、现代物流、工业设计等生产性服务业，提高产业配套能力。持续提升第五届中俄（佳木斯）农机展规模层次，带动发展会展业。加快金融改革，创新金融产品，扩大有效需求，支持涉农金融、保险机构发展。广泛建立友城关系，促进互惠合作。在佳天国际农副产品物流交易中心筹建海关监管仓库。加强口岸建设，发挥对俄合作优势，扩大进口资源落地加工、出口产品就地加工比重，吸引国内企业在佳木斯建设对俄合作总部或基地。鼓励引导我市农机、建材等领域企业到俄罗斯远东地区发展跨境连锁加工业。外贸进出口额同比增长8%以上。

（五）统筹兼顾，完善城乡服务功能。以居民需求为导向，推进城市建设管理提档升级。继续拓宽完善城区骨架路网，进一步修缮背街巷路。下决心解决群众反映强烈的违章建筑、占道经营、环境污染等问题，扎实开展裸地治理、“三废”出城和既有建筑节能改造工作。持续推进城区“三供三治”，新增城市日供水量2万吨，依法取缔城市供水管网覆盖范围内自备井；新增集中供热面积150万平方米，并网小锅炉12座；扩大城市供气规模，新安装天然气居民用户1.2万户；落实清雪“门前三包”制度，提高城区主次干道机械化清雪能力。加快推进老旧小区改造，着力解决最冷、最破楼房维修问题，提高小区及楼道亮化率。遵循规律，因势利导，健康有序地推进新型城镇化建设。加快户籍制度改革，放开农民进城落户限制，推进农村转移人口市民化，鼓励农民向城市、乡镇和中心村流动。重点加快7个中心镇、30个中心村整乡（镇）整村城镇化建设，完善基础设施和公共服务设施，促进产业和人口同步集聚。全面完成灾后城镇和农村居民点恢复重建及道路、给排水、环境绿化等基础设施建设。加快建立系统完善的生态文明制度体系，进一步释放生态发展潜能，形成绿色发展新优势。

（六）加大力度，构建民生幸福城市。（1）健全面向全体劳动者的职业培训制度，全市培训城乡劳动者6万人以上，开展职业技能鉴定并发证8000人次以上。（2）落实和完善更加积极的就业政策。加大小额担保贷款和劳动密集型小企业贷款力度，扶持全民创业。做好以高校毕业生为重点的就业援助工作。全年实现城镇新就业6.6万人以上，城镇登记失业率控制在4.5%以内。（3）转移农村劳动力36.5万人次，实现劳务收入36.5亿元以上。（4）养老保险、医疗保险、失业保险参保实现扩面，新农合参合率稳定在99.7%。（5）建立低保居民家庭信息共享机制，保证低收入群体享受政策机会均等公平。按照不低于去年我市城乡居民人均消费支出的30%确定最低生活保障标准。（6）加快推进养老服务，建设佳木斯老年康复养老中心。扶持和鼓励民间资本兴建养老机构。推进社区养老服务，探索居家养老方式。加快五保供养机构建设，集中供养能力达到65%以上。制定失能、半失能老人资助办法。（7）全市新建公共租赁住房1220套，新增租金补贴2050户，棚户区改造面积47.3万平方米。完成泥草房改造8800户。（8）有序推进中小学校网点布局调整。加强中小学校标准化、信息化建设，实施校长教师培训交流轮岗、强校带弱校制度，缩小城乡、校际差距，推动义务教育均衡发展。加强学前教育，严格执行义务教育学区制入学、中小学校阳光招生、阳光分班，促进教育公平。（9）引进高端医疗技术，打造专科医院特色品牌。探索社区医疗新模式，形成小病在社区、大病上医院、康复回社区的诊疗格局。推进乡镇卫生院、村卫生室一体化管理，增加专业全科医生配备数量。城乡居民电子健康档案建档率提高到75%。（10）实施文化惠民工程，大力发展文化产业。推动基层公共文化体育设施资源共建共享，广泛开展群众性文化、体育活动。（11）解决农村饮水困难与饮水安全3.6万人。城市饮用水源水质达标率100%。（12）提高环卫工人工资标准。（13）推进业主委员会建设，加强居民小区物业管理。推广弃管楼房社区服务站管理模式。（14）加强出租、客运车辆市场监管，严厉打击黑车、非法机动车扰乱客运市场行为。严格规范出租车油改气标准和质量检测，确保安全。（15）推进九龙、佳运公交重组工作，规划调整公交线路，增加、更新车辆，方便百姓乘车。（16）推进“平安佳木斯”建设，加大“两抢一盗”等犯罪行为打击力度。落实安全责任制，突出抓好安全隐患排查和治理，坚决遏制重特大安全事故发生。（17）推行网上受理信访制度，健全及时就地解决群众合理诉求机制。落实排查调处挂牌督办、领导干部接访下访、包案和责任追究制度，有效解决信访热点和遗留问题。（18）健全食品药品安全监管机制，建设“食品安全放心城市”。（19）全面推进城区社区管理体制改革，构建扁平化管理模式，提高社区工作者工资待遇和办公经费标准，改善信息化办公条件，解决70%的居委会办公用房。城乡标准化社区达标率70%以上。加快推进农村社区建设全覆盖。（20）稳步推进事业单位改革，加快解决居民回迁、房屋产权证照办理等历史遗留问题。

（七）转变职能，打造人民满意政府。以建设法治政府和服务型政府为目标，深化行政体制改革，创新行政管理方式，增强政府的公信力和执行力。推进简政放权。加大行政审批和行政权力清理力度，坚决取消一切不符合发展要求的审批事项，最大限度地简政放权，统筹兼顾地衔接运行，推进在市场经济中政府职能归位。对保留的行政审批事项和行政权力及时公开目录，精简优化流程，推行网上审批，切实提高审批效率和服务水平。启动市行政服务及公共资源交易中心，规范政府采购和资源交易行为。完善政务网站建设，依法公开政府权力运行流程，与民生密切相关的部门建立政务微博，搭建与群众交流平台。坚持依法行政。严格按照法定权限和程序行使权力、履行职责，科学民主决策，自觉接受人大、政协监督，重视司法、舆论和社会公众监督。清理规范中介组织，打破中介机构垄断，中介机构与主管部门全部脱钩。强化对政府部门履职尽责情况的效能监察，严格问责失职渎职行为。加强廉政建设。深入开展群众路线教育实践活动，健全完善反对“四风”的制度体系和业务规范。改进预算管理制度，强化预算刚性，政府及各部门预决算、各部门三项经费预决算向社会公开。防范政府债务风险，规范政府投资项目管理。制定大项目资金预算管理办法，政府重大投资和支出项目审计实现全覆盖，有效预防、坚决惩治腐败，促进干部清正、政府清廉、政治清明。

各位代表，时代赋予重任，奋斗铸就辉煌。我们要倍加珍惜来之不易的发展态势，倍加珍惜团结和谐的大好局面，在市委的正确领导下，以更加高昂的斗志、更加扎实的举措、更加务实的作风，解放思想，开拓创新，拼搏奋进，为全面完成市委全会确定的各项目标、加快建设富裕文明和谐幸福佳木斯而努力奋斗！
